# Fracture strength of orthodontic mini-implants

**DOI:** 10.1590/2177-6709.22.3.047-054.oar

**Published:** 2017

**Authors:** Tatiana Feres Assad-Loss, Flávia Mitiko Fernandes Kitahara-Céia, Giordani Santos Silveira, Carlos Nelson Elias, José Nelson Mucha

**Affiliations:** 1 Universidade Federal Fluminense, Department of Orthodontics (Niterói/RJ, Brazil).; 2 Instituto Militar de Engenharia, Materials Engineering, Biomaterials Laboratory, Rio de Janeiro, RJ, Brazil.

**Keywords:** Orthodontics, Skeletal anchorage, TADs, Mini-implant, Dental materials.

## Abstract

**Objective:**

This study aimed at evaluating the design and dimensions of five different brands of orthodontic mini-implants, as well as their influence on torsional fracture strength.

**Methods:**

Fifty mini-implants were divided into five groups corresponding to different manufactures (DEN, RMO, CON, NEO, SIN). Twenty-five mini-implants were subjected to fracture test by torsion in the neck and the tip, through arbors attached to a Universal Mechanical Testing Machine. The other 25 mini-implants were subjected to insertion torque test into blocks of pork ribs using a torquimeter and contra-angle handpiece mounted in a surgical motor. The shape of the active tip of the mini-implants was evaluated under microscopy. The non-parametric Friedman test and Snedecor’s F in analysis of variance (ANOVA) were used to evaluate the differences between groups.

**Results:**

The fracture torque of the neck ranged from 23.45 N.cm (DEN) to 34.82 N.cm (SIN), and of the tip ranged from 9.35 N.cm (CON) to 24.36 N.cm (NEO). Insertion torque values ranged from 6.6 N.cm (RMO) to 10.2 N.cm (NEO). The characteristics that most influenced the results were outer diameter, inner diameter, the ratio between internal and external diameters, and the existence of milling in the apical region of the mini-implant.

**Conclusions:**

The fracture torques were different for both the neck and the tip of the five types evaluated. NEO and SIN mini-implants showed the highest resistance to fracture of the neck and tip. The fracture torques of both tip and neck were higher than the torque required to insert mini-implants.

## RESUMO

**Objetivo:**

o objetivo do presente estudo foi avaliar o desenho e as medidas de cinco marcas diferentes de mini-implantes ortodônticos, e sua influência na resistência à fratura em torção.

**Métodos:**

cinquenta mini-implantes foram divididos em cinco grupos, correspondentes a diferentes fabricantes (DEN, Dentaurum; RMO, Rocky Mountain Orthodontics; CON, Conexão; NEO, Neodent; SIN, Sistema de Implantes Nacional). Vinte e cinco mini-implantes foram submetidos ao teste de fratura por torção no pescoço e na ponta, com mandris fixados a uma máquina universal de testes mecânicos. Os outros 25 mini-implantes foram submetidos ao teste de torque de inserção em blocos de costelas suínas, utilizando-se um torquímetro e um contra-ângulo montado em motor cirúrgico. O formato da ponta ativa dos mini-implantes foi avaliado por microscopia. O teste não-paramétrico de Friedman e o teste F de Snedecor na análise de variância (ANOVA) foram utilizados para avaliar as diferenças entre os grupos.

**Resultados:**

o torque de fratura do pescoço variou de 23,45 N.cm (DEN) a 34,82 N.cm (SIN); e o da ponta, entre 9,35 N.cm (CON) e 24,36 N.cm (NEO). O valor do torque de inserção variou de 6,6 N.cm (RMO) a 10,2 N.cm (NEO). As características que mais influenciaram os resultados foram: diâmetro externo, diâmetro interno, razão entre o diâmetro interno e o externo, e a presença de fresagem na região apical do mini-implante.

**Conclusões:**

os torques de fratura foram diferentes entre os cinco tipos avaliados, tanto no pescoço quanto na ponta. Os mini-implantes NEO e SIN foram os mais resistentes à fratura do pescoço e da ponta. Os torques de fratura tanto na ponta quanto no pescoço foram maiores do que o torque necessário para a inserção dos mini-implantes.

## INTRODUCTION

Mini-implants have been an effective anchorage method,^1^ being very well tolerated by patients,^2^ creating new possibilities for orthodontic treatment that requires minimal cooperation and maximum aesthetics results, particularly in adults.^3^ Unlike osseointegrated dental implants made of commercially pure titanium, mini-implants are manufactured with the alloy Ti6Al4V (ASTM grade 5).^4^ ^,^ ^5^ This alloy has higher mechanical strength than pure titanium^6^ and it is best suited to the small diameter of mini-implants. It also presents bioactive characteristics inferior to pure titanium, thus facilitating removal because it promotes lower osseointegration.^4^ ^,^ ^5^

The mini-implant installation is simple, and it can be inserted in various sites because of its reduced size,^1^ ^,^ ^7^ ^,^ ^8^ including those between the roots of teeth using mono or bicortical anchor,^9^ alone or connected by an interchangeable structure.^10^ On the other hand, the small size of mini-implants empowers an increase of fracture during insertion, deformation or fracture during its removal.^4^ Bicortical anchorage adds a new challenge to the fracture resistance of orthodontic mini-implants especially in the tip region, which has a smaller diameter. Another risk of fracture could occur when mini-implants are placed in the external oblique ridge area of the mandible because of the thickness and hardness of the cortical bone in this area.^10^

Mini-implants can be found in the market with different shapes, designs, diameters, lengths, degree of titanium alloy purity and surface treatments.^5^ However, mini-implants with similar dimensions have different design that may influence the fracture resistance and are relied upon by the various manufacturers to improve clinical performance.^6^ ^,^ ^11^ The fracture resistance of the mini-implants is a decisive aspect to assist the clinician in choosing the most appropriate and safe device.

Therefore, the main objective of this study was to evaluate the design, to measure the insertion torque and to quantify the maximum fracture torque resistance of five different orthodontic mini-implants. 

## MATERIAL AND METHODS

Fifty orthodontic self-perforating mini-implants from five different manufacturers (DEN: Dentaurum, Ispringen, Baden-Württemberg, Germany; RMO: Rocky Mountain Orthodontics, Seoul, South Korea; CON: Conexão, Arujá, São Paulo, Brazil; NEO: Neodent, Curitiba, Paraná, Brazil; and SIN: Sistema de Implantes Nacional, São Paulo, São Paulo, Brazil) similar in size dimension, were divided into five groups, as shown in Tables 1 and 2.

**Table 1:** Groups code, milling in the apical region, trade name, manufacturer, source, chemical composition, nominal diameter, length and transmucosal profile of mini-implants used in the study.

Groups	Apical milling	Trade name	Manufacturer and lot	Origin	Chemical composition	Diameter (mm)	Length (mm)	Profile (mm)
DEN	Yes	Tomas Ref 302-106-10	Dentaurum 394727	Ispringen, Germany	Ti_6_Al_4_V	1.6	6	ND*
RMO	Yes	Dual-top Anchor System Ref Goo213	Rocky Mountain Orthodontics 022367	Seoul, South Korea	Ti_6_Al_4_V	1.6	6	ND*
CON	Yes	Ortoimplante Ref P9900099	Conexão 8081468146	Arujá, SP Brazil	Ti_6_Al_4_V	1.5	6	1
NEO	No	Implante Ancoragem Ortodôntica Ref 109496	Neodent 2788897	Curitiba PR, Brazil	Ti_6_Al_4_V	1.6	7	1
SIN	No	Wire Dynamic Ref POTC 1616	SIN - Sistema de Implantes Nacional F60556	São Paulo SP, Brazil	Ti_6_Al_4_V	1.6	6	1

*ND = Not disclosed.

**Table 2:** Values of linear measurements (µm) and angle (degrees) of mini-implants.

Feature evaluated	Groups				
	DEN	RMO	CON	NEO	SIN
Length of tip	6.006,01	5,329.59	5,926.47	6,812.51	6,090.93
Outside diameter	1.607,96	1,539.77	1,482.95	1,630.72	1,562.59
Inside diameter	1.079,55	1,028.42	772.75	1,107.97	1,164.77
Number of threads	6	7	12	9	7
Step of threads	888,09	735.39	464.49	732.32	756.49
Angle of the screw thread (degrees)	140,22	137.69	128.85	135.31	128.46
Length of the thread franc	381,96	384.78	232.11	360.58	292.79
Length from the bottom of the screw thread	395,63	327.65	239.77	323.86	376.89
Taper (ratio= b-a/2xD)	0,11	0.07	0.09	0.1	0.1
Percentage between diameters	67%	67%	52%	68%	75%

**Figure 1:**
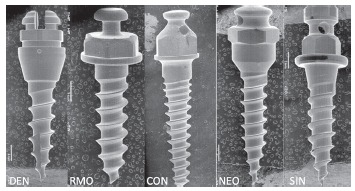
Microphotograph illustrating the superficial aspects and superficial finish quality of five types of mini-implants used (DEN, RMO, CON, NEO, SIN).

The surface morphology and chemical composition of the mini-implants in each group was analyzed by scanning electron microscope (SEM; Jeol JSM-5800LV) at 20kV and observed at 500x magnification and 50 µm away, by energy dispersive spectroscopy (EDS). Figure 1 illustrates SEM microphotographs of the mini-implants for each group. 

### Mini-implants design evaluation

**Figure 2:**
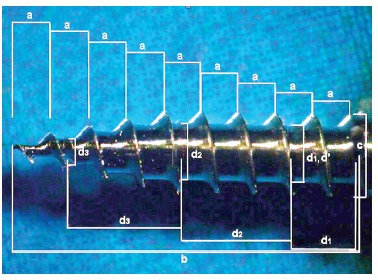
Linear measurements made in pictures of the mini-implants in optical microscope with an increase of 1.6 x. Pitch of the threads (a), total length or active tip of the mini-implant (b), the external diameter of mini-implant (c), steps for calculating the taper of the mini-implant (d), and internal diameter of the mini-implant (d’).

**Figure 3:**
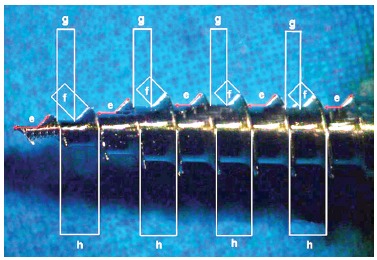
Linear and angular measurements made in pictures of the mini-implants in optical microscope with an magnification of 1.6 x. Angle of the screw thread (e), the free length of thread (f), and the length of the bottom fillet of the thread (g) of the screw thread pitch (h).

Pictures of the mini-implants were obtained under optical microscope (Zeiss Stemi 2000-C, Zeiss) observed at 1.6 x magnification. The pictures of the surfaces were analyzed with Axio Vision program (Zeiss) which were made of linear and angular measurements, as shown in Figures 2 and 3.

### Mini-implant insertion torque

Six frozen pig ribs, from the same animal, were prepared to be used as material for insertion of the mini-implants. After slaughter, the ribs were cleaned to remove tissue and stored in normal saline at 4°C for 24 hours. Then, they were cut transversely to obtain 60 bone blocks of 2 x 2 cm and stored again in physiological saline at 4°C until assay insertion occurred between the first and the third day.

Each pork rib bone block was randomly picked among the 60 bone blocks and was attached to an adjustable piece of metal, for size and shape standardization. This piece of metal was attached to the digital torque meter (Lutron TQ-8800 torque meter) connected to a computer and pre set to prevent any movement during the test.

**Figure 4:**
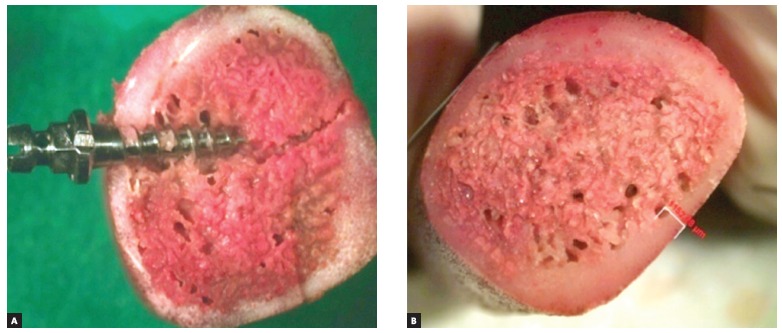
Mini-implant position in pork rib (A) and cortical bone thickness of pork rib block used in the insertion test of mini-implants (B).

Tests on insertion of the mini-implant were carried out without performing any preliminary drilling, using the mini-implant coupled to the switch for short contra angle for each specific group of mini-implants, fitted to a hand piece with reduced speed (20:1) at 50 rpm (Anthogyr Instruments) with MC -101 surgical drill, and Omega II Dentscler motor. Each mini-implant was inserted in the block located in the central portion of pork ribs perpendicular to the cortical bone (Fig 4).

The same calibrated operator performed the whole assay. Initial tests were undertaken to verify the procedure and to maintain the strength standardized in relation to the long axis of the mini-implant. The insertion torques were recorded continuously by the Lutron 101 program, version V0011TW (Lutron Electronic Enterprise). 

Maximum and minimum insertion torque values were considered as the largest and the smallest values, respectively. Each bone block, properly identified, was taken to the Optical Zeiss Stemi 2000-C Microscope (Zeiss) with 1.6x and 5x magnification for measurement of cortical thickness of the bone at the site of insertion of the mini-implant. The images were captured in the cortical computer and sent to the Axio Vision program (Zeiss) where their thicknesses were measured.

## MINI-IMPLANT FRACTURE TORQUE

Five mini-implants in each group (DEN: Dentaurum, RMO: Rocky Mountain Orthodontics, CON: Conexão, NEO: Neodent; SIN: Sistema de Implantes Nacional) were submitted to fracture torsion testing. The maximum torque fracture was determined in the lower third of the active tip (tip) and the upper third of the active tip (neck). For this test, a device with two mandrels coupled to universal mechanical testing machine (EMIC) with a 500N load cell was used.

In the fracture test, the key for short contra-angle surgical kit, specific for each group coupled to the head of the mini-implant, was attached to the mandrel, and the other mandrel was attached to the other end of the mini-implant. The left mandrel rotated by traction of a polymer strand attached to the shaft of the testing device and the load cell, thereby applying a torque on the mini-implant, once the right mandrel was fixed.

In the first mechanical testing, the fracture torque of the mini-implant tip was measured. In the second fracture testing, the maximum fracture torque of the region close to the neck was determined.

In order to calculate the fracture torque (N.cm) the maximum force was obtained from records of the Universal Mechanical Testing Machine (EMIC), based on the following formula: T = F x 0.4, where T = Torque (N.cm), F = Force (N), and 0.4 cm is the radius of the shaft on which the polymer strand was wound.

## STATISTICAL ANALYSIS

The numerical results were presented as means and standard deviations. The non-parametric Friedman test was used to assess torque values of fracture of the tip and the neck of the mini-implant. Snedecor’s F in analysis of variance (ANOVA) was used to evaluate the insertion torque and the thickness of cortical bone. The data obtained from all measurements were processed with SPSS software, version 13.0 (IBM, Chicago, Illinois, USA). The level of significance for all statistical tests was predetermined at 1%.

## RESULTS

Table 2 presents the results of mini-implants design and the measures corresponding to the length of tip, outer diameter, inner diameter, and the number of threads (Figs 2 and 3).The bone material used for the test was the pork rib, which has been used in other studies, as the thickness of cortical bone is similar to that found in the human jaw, around 0.5 to 1.0 mm.^12^ In this study the cortical thickness of the pork rib blocks showed a thickness average from 1.30 to 1.52 mm and no significant difference among groups was determined (Table 3). Therefore, it can be assumed that the bone material used to insert the mini-implants did not influence the results. The average and standard deviations of the torque during insertion in the five groups and the average values and standard deviations for the thickness of the cortical bone blocks used as material for insertion were calculated (Table 3).

**Table 3:** Means and standard deviations (SD) of the insertion torque (N.cm) and cortical bone thickness (mm), Snedecor’s F test in ANOVA.

Groups	Insertion		Cortical thickness	
	Mean ± SD	F test, p	Mean ± SD	F test, p
DEN	7.80 ± 1.30^AB^	F = 4.66 p = 0.008*	1.52 ± 0.38	F = 0.43 p = 0.786 ns
NEO	10.20 ± 0.84^A^		1.50 ± 0.17	
SIN	8.20 ± 1.79^AB^		1.30 ± 0.35	
CON	7.40 ± 1.34^B^		1.30 ± 0.25	
RMO	6.60 ± 1.52^B^		1.47 ± 0.56	

Same letters: no difference. Different letters: significant differences.

* = Significant (p < 0.01) NEO X CON, p = 0.015, NEO x RMO, p = 0.013.

ns = Non significant (p > 0.01).

The torque values of fracture on the neck region were higher than the torque values to obtain a fracture in the region of the tip in all groups (Table 4). 

**Table 4:** Means and standard deviations (SD) of fracture torque (N.cm) in the neck and tip of the five groups, tested with Snedecor’s F in ANOVA and XR^2^ non-parametric “Friedman” for comparison between groups (p < 0.01).

	Site							
Groups	NECK				TIP			
	Mean	SD	Test	Sig.	Mean	SD	Test	Sig.
DEN	23.45^A^	3.08	F= 9.04	0	10.56 ^A^	5.12	Xr^2^= 17.53	0.002
NEO	27.28 ^A^	0.98			24.36 ^B^	2.58		
SIN	34.82 ^B^	3.5			22.88 ^B^	2.98		
CON	25.70 ^A^	5.01			9.35 ^A^	3.22		
RMO	32.41 ^AB^	3.5			21.95 ^B^	2.79		

Same letters: no difference. Different letters: significant differences.

* = Significant (p < 0.01) DEN X SIN, p = 0.014, NEO x SIN, p = 0.011.

ns = Non significant (p > 0.01).

## DISCUSSION

The mini-implants diameter showed a variation from 1,630.72 µm (NEO) to 1,482.95 µm (CON). The CON group, besides having the smallest diameter, also showed a lower torque value of fracture of the tip (9.35 N.cm) and the second lowest value of fracture torque on the neck (25.7 N.cm). A similar result was observed in mini-implants with a diameter less than 1.5 mm, which were more susceptible to fracture.^13^ ^,^ ^14^ ^,^ ^15^

The torque values of fracture on the neck and the tip region could indicate that the diameter of the fracture site is an important variable in fracture torque, because the small-diameter mini-implant is an important risk factor for fractures, especially in insertion or removal time.^1^ ^,^ ^4^ ^,^ ^8^ ^,^ ^14^ ^,^ ^15^ ^,^ ^16^ ^,^ ^17^ Although mini-implants with a reduced diameter have increased risk of fracture, the choice of mini-implants with a diameter that is too large can lead to bone necrosis through micro fractures in bone, and dental structures risks.^15^ ^,^ ^16^

In relation to the tip region, the lowest torque value of fracture was found in the CON group (9.35 N.cm), which was statistically significant (*p* <0.01) compared with the NEO (24.36 N.cm), SIN (22.88 N.cm), and RMO (21.95 N.cm) groups. These differences may be a consequence of the great difference among them in shape, in ratio of external to internal diameter and in the number of threads, and consequently, in the step of threads.^18^

CON group presented the second lowest value of fracture torque in the neck (25.7 N.cm), and it is statistically significant (*p <* 0.01) when compared with SIN (34.82 N.cm), which showed highest value of fracture torque. Additionally, when the internal diameter of mini-implant between these two groups was compared, great variation was found. The CON group exhibited the lowest mean (772.75 µm), while the SIN group presented the highest (1164.77 µm). The inner diameter of the mini-implant is an important characteristic of susceptibility to fracture.^19^

Furthermore, the mini-implants in the CON group also showed the smallest external diameter (1482.95 µm), and a greater number of threads.^7^

The taper of CON group was smaller, which means it was a cylinder mini-implant. Mini-conical implants would be most suitable because it could have the thickness thinner and a diameter more resistant immediately below the point of loading application.^20^ However, fractures occur less frequently during application of orthodontic force and more often during insertion or removal of the mini-implants.^8^ In the present study, the tip region was the most susceptible area to fracture.

The results showed that SIN and NEO exhibited the highest mean fracture resistance as referred elsewhere.^21^ The lower fracture torque on the neck was found in DEN (23.45 N.cm). In the tip region, the DEN presented the second lowest value of fracture torque (10.56 N.cm). Possibly this was due to differences in relation to the steps of thread and taper. The DEN group had means values of thread steps and taper larger than the other groups, contradicting the fact that fracture strength can be increased with the conical design.^22^

The lowest torque fracture values was shown in the tip region of CON and DEN groups and were also the groups that had the apical milling. The presence of this lateral groove reduces the internal diameter of the narrower region of the mini-implants which probably increased the fracture susceptibility at the tip, which would justify the lower values of fracture torque found in these groups.

Mini-implants of these five groups were made with the Ti6Al4V alloy, as perceived by energy dispersive spectroscopy. However, the strength of the titanium alloy depends also on the microstructure, which is influenced by the composition, heating treatment, and thermo-mechanical processing of the mini-implants.^6^ These variables must be evaluated in further studies.

The insertion torques ranged from 6.6 N.cm (RMO) to 10.2 N.cm (NEO). All groups had values that were within the recommended boundary^24^ ^,^ ^28^ between 5 and 10 N.cm, and the values of insertion torque could reach 15N.cm without major problems. All fracture torque resistance were higher than the insertion torque values in both the fracture tip region and the neck in all groups, showing that all mini-implants tested can be considered safe from the risk of fracture in clinical use. The insertion values showed significant differences among NEO (10.2 N.cm), CON (7.4 N.cm) and RMO (6.6 N.cm). The difference in insertion torque among the groups was possibly due to the difference in design among the mini-implants. The mini-implants designs showed a large difference between them in relation to the number of threads of screws, and consequently the pitch of the thread.

The NEO group had the second highest number of threads (n = 9) and was the group with the highest insertion torque (10.20 N.cm). As the retention mechanism is based on fitting mechanical structure in cortical and not necessarily the concept of osseointegration,^4^ ^,^ ^5^ the shape and length of the screw threads are fundamental to primary stability.^20^ ^,^ ^22^ ^,^ ^25^ Greater number of threads and closeness between them increase the mechanical stability and resistance in the insertion in the bone.^5^ However, there were significant differences between NEO and CON groups, which had the largest number of threads,^7^ and the second lowest value of insertion torque (7.4 N.cm). This result was probably due to the difference in diameter between these two groups. The diameter is significantly associated with stability.^16^ NEO and CON groups showed the highest (1,630.72 µm) and lowest (1,482.95 µm) outer diameter, respectively, among all groups. The outer diameter appears to be an important feature of variation of the insertion torque.^26^ NEO group, with the largest diameter (1,630.72 µm), had the highest value of insertion torque (10.20 N.cm), and RMO and CON groups, with smaller diameters, showed the lowest values.

During the insertion, control features should be used such as micro-motor with controlled torque,^13^ manual wrench^1^ or dynanometer^27^ to prevent this torque from approaching or reaching the fracture torque of mini-implants. Fracture toughness of mini-implants varies according to the manufacturer and type of mini-implant, so the operator should be aware of the characteristics of mini-implants that influence the torque values of fracture before choosing one to be used, and the maximum torque that can safely be used clinically for the insertion.^18^

## CONCLUSIONS

Mini-implants of different brands have different design and morphology, and fracture torques resistance is determined by: outside diameter, internal diameter, ratio of inner and outer diameter, and milling in the apical region.

Among the mini-implants trademark evaluated, NEO and SIN mini-implants have showed the highest resistance to fracture of the neck and tip.

The insertion values torques was always lower than the fracture torque resistance of both the tip and the neck in all groups tested.
